# Knowledge and perception towards net care and repair practice in Ethiopia

**DOI:** 10.1186/s12936-017-2043-1

**Published:** 2017-10-02

**Authors:** Ayele Zewde, Seth Irish, Adugna Woyessa, Yonas Wuletaw, Honelgn Nahusenay, Semira Abdelmenan, Meaza Demissie, Hanna Gulema, Gunawardena Dissanayake, Sheleme Chibsa, Hiwot Solomon, Meseret A. Yenehun, Amha Kebede, Lena M. Lorenz, Gabriel Ponce-de-Leon, Joseph Keating, Alemayehu Worku, Yemane Berhane

**Affiliations:** 1 Addis Continental Institute of Public Health, Addis Ababa, Ethiopia; 2 Entomology Branch, Division of Parasitic Diseases and Malaria, Center for Global Health, Centers for Disease Control and Prevention and U.S. President’s Malaria Initiative, Atlanta, USA; 3 Ethiopian Public Health Institute, Addis Ababa, Ethiopia; 4 President Malaria Initiative (PMI-USAID), Addis Ababa, Ethiopia; 5 Ethiopian National Malaria Prevention, Control and Elimination Program, Addis Ababa, Ethiopia; 6 London School of Hygiene and Tropical Medicine, London, UK; 7 Centers for Disease Control and Prevention, Atlanta, USA; 8 School of Public Health and Tropical Medicine, Tulane University, New Orleans, USA

**Keywords:** Net care and repair, Knowledge, Perception, LLIN, Ethiopia

## Abstract

**Background:**

Long-lasting insecticidal nets (LLINs) are a key malaria control intervention. Although LLINs are presumed to be effective for 3 years under field or programmatic conditions, net care and repair approaches by users influence the physical and chemical durability. Understanding how knowledge, perception and practices influence net care and repair practices could guide the development of targeted behavioural change communication interventions related to net care and repair in Ethiopia and elsewhere.

**Methods:**

This population-based, household survey was conducted in four regions of Ethiopia [Amhara, Oromia, Tigray, Southern Nations Nationalities Peoples Region (SNNPR)] in June 2015. A total of 1839 households were selected using multi-stage sampling procedures. The household respondents were the heads of households. A questionnaire was administered and the data were captured electronically. STATA software version 12 was used to analyse the data. Survey commands were used to account for the multi-stage sampling approach. Household descriptive statistics related to characteristics and levels of knowledge and perception on net care and repair are presented. Ordinal logistic regression was used to identify factors associated with net care and repair perceptions.

**Results:**

Less than a quarter of the respondents (22.3%: 95% CI 20.4–24.3%) reported adequate knowledge of net care and repair; 24.6% (95% CI 22.7–26.5%) of the respondents reported receiving information on net care and repair in the previous 6 months. Thirty-five per cent of the respondents (35.1%: 95% CI 32.9–37.4%) reported positive perceptions towards net care and repair. Respondents with adequate knowledge on net care and repair (AOR 1.58: 95% CI 1.2–2.02), and those who discussed net care and repair with their family (AOR 1.47: 95% CI 1.14–1.89) had higher odds of having positive perceptions towards net care and repair.

**Conclusions:**

The low level of reported knowledge on net care and repair, as well as the low level of reported positive perception towards net repair need to be addressed. Targeted behavioural change communication campaigns could be used to target specific groups; increased net care and repair would lead to longer lasting nets.

**Electronic supplementary material:**

The online version of this article (doi:10.1186/s12936-017-2043-1) contains supplementary material, which is available to authorized users.

## Background

Malaria continues to be one of the biggest public health problems in Ethiopia; 75% of the country’s land mass is considered to be malarious and 60% of the population resides in these areas [1, 2]. According to the 2011 Malaria Indicator Survey (MIS), around 77% of cases were due to *Plasmodium falciparum* and the remainder was *Plasmodium vivax* [3]. The long-lasting insecticidal net (LLIN) is a key malaria control intervention, and is currently a priority for scale-up in areas where malaria transmission occurs. If properly used, LLINs act not only as a physical barrier against biting mosquitoes, but also substantially reduces malaria transmission [4–7].

As in most countries in sub-Saharan Africa, LLINs are an important tool for malaria prevention and control in Ethiopia [8]. According to the 2011 MIS, 55% of households reported owning at least one LLIN [3], with varying levels of net ownership across regions in Ethiopia [9, 10]. The level of LLIN utilization also showed variation by region in Ethiopia [3]. According to the World Health Organization Pesticide Evaluation Scheme (WHOPES), LLINs are expected to remain effective for 3 years under field conditions [11]. However, varying levels of durability in the field have been reported [12–14], with only a few studies confirming their usefulness over the entire 3 years [15, 16].

The duration of physical integrity and chemical effectiveness of LLINs are often influenced by factors such as household condition, frequency of washing, type of cooking fuel used, the location of the kitchen inside the house, net repair practice [17, 18], and the level of care given to nets in general [19–21]. Increased net care and repair practices could increase the longevity of the net: keeping the net away from children, pests and rodents; rolling up the net when not in use; and washing the net gently were some of the recommended net care approaches [19–21]. In addition, it is also recommended to repair any small hole in the net immediately [22]. Repairing small holes immediately could prolong the physical durability of the LLIN, although hole repair practices are uncommon [23, 24].

For households to employ net care and repair practices, knowing how to adequately care for and repair a net is the starting point. Given the variation in what is reported as net care and repair [21, 22, 25], it is important that knowledge is assessed contextually. Perception towards net care and repair practice (e.g., holes can be fixed, fixing holes will make the net last longer, others in the community are also fixing holes, and confidence in one’s ability to repair a net) is also important, as behavioural change processes are influenced by perception [21].

Although, many studies have systematically assessed issues related to LLIN longevity, there is little evidence on knowledge and perception towards net care and repair in Ethiopia. The purpose of this study is to measure the level of knowledge about net care and repair approaches, identify perceptions towards net care and repair, and isolate factors that influence these perceptions among households in Ethiopia.

## Methods

### Study site

This study was conducted in four regional states of Ethiopia: Amhara, Oromia, Southern Nations Nationalities Peoples Region (SNNPR), and Tigray. About 86% of the population of Ethiopia inhabit these regions [26]. The overall prevalence of malaria in the study site was between 0.7 and 1.3% in areas below 2000 m; SNNPR had the highest prevalence (2.5%) followed by Amhara (2.0%), while Oromia (0.5%) and Tigray (0.6%) had lower prevalence [3]. Only 23% of the households in these regions have access to electricity, 70.1% have only one room for sleeping, and 52.5% cook inside the main house, 77.0% use wood fire as energy source. Regarding education, 50.8% of women and 29.5% of men did not attend formal education [27].

### Study design

This is a cross-sectional baseline survey, which is part of a large, longitudinal, multi-site study designed to enrol and monitor cohorts of nets across four regions of Ethiopia over 3 years to assess physical and chemical durability. This study was carried out following the mass LLIN campaign conducted in 2015.

### Sample size and sampling procedure

The sample size for the baseline survey was calculated following the WHO standard for phase III filed trial of nets [11]. The calculation yielded 460 households for each region, by assuming 95% confidence interval and 80% power, and a net attrition rate of 20% per year and 50% over 3 years. The total sample size for the four regions was 1840 households. Each region constituted a survey domain. A three-stage sampling procedure was used to select households from each region. (1) Districts were defined to belong to low, moderate or high malaria transmission areas. Only districts where the LLINs distribution campaign had already been completed were included in the study. These were identified in consultation with Regional Health Bureau. At the time of the survey, LLIN distribution had taken place in only two and three districts in the low and high transmission areas, respectively. All five districts were included. To select districts from the moderate transmission areas, Excel random generation was used to select seven districts out of 30 eligible districts; (2) Clusters [enumeration areas (EAs) containing 150–200 households] were selected using simple random sampling. To get the required sample size for each region (460) and because 20 households were to be selected for each cluster (see below), a total of 23 clusters were selected across the districts. Clusters were allocated proportionally to the size of the population in each district. On average, eight clusters were selected in each district; and, (3) 20 households were selected from each cluster using systematic random sampling. Data collectors used a household listing and sampling sheets to select the 20 households. All the names of the heads of the households, their receipt of LLINs during the 2015 distribution campaign and their presence at home on one of two visit attempts were recording on the sampling sheets. Those households that fulfilled the inclusion criteria were given a sampling number. To get the sampling interval (K), the total number of households included was divided by 20. To start the sampling, a random number was generated between 1 and K using a simple lottery method and every Kth household in the sample was visited. The head of household or an adult member of the household (aged 18 years or above) was interviewed.

### Data collection procedure

Data were collected electronically using a structured questionnaire approximately 2 months after LLINs were distributed to households. Questions on household characteristics, socio-demographic factors, knowledge related to net care, exposure to information on net care and repair, and perceptions of net care and repair were asked. The questionnaire was pre-tested in advance of data collection.

Trained data collectors and supervisors conducted data collection using a hand-held tablet device with electronic questionnaire developed using an Open Data Kit (ODK) program [28]. Supervisors reviewed the data and sent them to the server at Addis Continental Institute of Public Health (ACIPH) daily, or as soon as internet connectivity allowed. The data management team at ACIPH downloaded and reviewed the data daily. The team provided feedback to the supervisors in the field in terms of completeness and errors to be fixed.

### Measurement

#### Socio-demographic variables

Educational status of the household head was classified as illiterate (person who cannot read and write), able to read and write, primary, secondary, and high school and above. Data on age were collected as a continuous variable and categorized in to groups using 5-year intervals. The economic status of the households was measured based on a composite measure wealth index based on household assets and house condition [29], then categorized into quintiles.

#### Perception-related variables

A series of eight Likert-scale statements were presented to the respondent to measure perception towards net care and repair. The responses were captured across a scale of 5, ranging from completely disagree to completely agree. Additional file 1 shows the eight perception statements used to capture perception towards net care and repair.

The response for each statement was coded as − 2 “completely disagree”, − 1 “disagree”, 0 “neutral”, 1 “agree”, or 2 “completely agree”. To calculate the overall perception score, the response to eight of the perception statements were added-up and divided by 8 to generate mean perception levels for everyone. Based on the mean score, respondents were further categorized as having negative perceptions when their score was ≤ 0; having positive perception when their score range was between 0.01 and 1.0; and, having very positive perception when their score was between 1.01 and 2.0 [19, 30].

#### Exposure to information on LLINs

Participants were asked if they have received information regarding LLINs in the 6 months prior to the survey and their response was coded as yes or no. Participants who said they had received information were then asked what the information was about. From the list of topics, the respondents could provide multiple responses. Participants were also asked if they had discussed net care and repair with their family; their response was captured as “yes” or “no”.

#### Knowledge on net care and repair

Participants were asked what action they would take to prevent holes. Their response was captured from the following list of actions: “keep away from children”; “keep away from pests and rodents”; “roll-up or tie-up when not in use”; “handle the net with care”; “do not soil with food”; “keep away from flames”; “wash gently”; “wash only when dirty”; “inspect regularly for holes”; “repair small holes quickly”. Respondents who stated five or more (e.g., over half) of the correct answers were dichotomized as having knowledge on net care and repair and the remaining as not having knowledge.

### Data analysis

Data analysis was done using STATA version 12 (Stata Corporation, College Station, TX, USA) using the “*surveyset”* command to account for complex survey data, population weights were also applied to account for unequal probability of selection across some districts. Descriptive statistics across outcomes are provided. Ordinal logistic regression was used to identify factors associated with the perception towards net care and repair. Factors tested include knowledge towards net care and repair, exposure to information on net care and repair, discussion on net care and repair in the family, and number of LLINs owned by the household. The model also controlled for the following socio-demographic variables: age of the respondent, gender, educational status, wealth, and region.

### Ethical consideration

The Institutional Review Board (IRB) at Addis Continental Institute of Public Health (ACIPH) approved the protocol. Permission letters were also obtained from each study region (Amhara, Oromia, Tigray, SNNP) and selected districts. At the household level, the study was fully explained to the respondent and a verbal consent was obtained from each participant.

## Results

### Socio-demographic characteristics of household respondent

A total of 1839 households were included in the sample and the response rate was 99.9%; only one house was excluded from the study. The majority of the respondents were male (80.2%) and head of the household (98.8%). The mean age of the respondents was 44 years and 24.8% were 55 years old and above. More than half of the head of the households (53.5%) reported not attending formal school, suggesting low level of literacy. The average household size was 5.17 persons and ranged from 1 to 12 individuals. More than 90% of the houses had floors made of earth and 76.5% of houses used corrugated iron for roofing material (Table 1).

**Table 1:** Socio-demographic characteristics of households and respondents, Ethiopia 2015

Variables	Percentage (%)	Standard error
Gender n = 1839		
Male	80.2	0.0096
Female	19.8	0.0096
Age n = 1839		
18–29	12.77	0.0821
30–39	30.02	0.0113
40–49	24.50	0.0107
50–59	14.09	0.0085
60+	18.62	0.0091
Mean ± SD		
44 ± 0.33		
Relationship with the household head n = 1839		
Head	98.8	0.0025
Wife of the head	1.1	0.0023
Son/daughter	0.06	0.0006
Grandchild	0.1	0.0008
Educational status of head of household^a^ n = 1839		
No formal education	53.70	0.0116
Primary (grade 1–6)	25.57	0.0102
Secondary (grade 7–8)	8.27	0.0064
More than secondary (≥ grade 9)	12.46	0.0077
Mean household size	5.17	0.5126
Wealth index n = 1829		
1st	20.1	0.0097
2nd	20.0	0.0099
3rd	20.1	0.0099
4th	19.9	0.0010
5th	20.0	0.0090
Region n = 1839		
Tigray	24.2	0.0012
Amhara	26.4	0.0009
Oromia	27.7	0.0016
SNNP	21.6	0.0010
Household characteristics		
Roofing n = 1839		
Grass/leaf	19.3	0.0073
Mud	1.1	0.0027
Rustic mat/plastic sheets	2.1	0.0033
Corrugated iron/wood	76.5	0.0082
Cement/concrete	0.7	0.0017
Stone	0.3	0.0014
Floor n = 1839		
Earth	91.7	0.0055
Wood/bamboo/palm	1.7	0.0026
Vinyl/parquet	0.004	0.0004
Tiles/cement	6.40	0.0006

^a^Education categories refer to the highest level of education attended, whether or not that level was completed

### Exposure to information and knowledge on net care and repair

A quarter of the respondents said they have received information on net care and repair in the 6 months prior to the survey. The most commonly reported topic was “hang-up your net” (72.8%; 95% CI 68.6–77.1%) followed by “care for your net” which was reported by 56.6% (95% CI 52.1–61.2%) of respondents. Only 3.0% (95% CI 1.5–5.1%) of the respondents reported receiving information on net repair. Health extension workers (HEWs) were the main source of net care and repair information. Approximately 19% of respondents reported discussing net care and repair with their family, after being asked about any such discussions. Respondents’ knowledge on net care and repair is presented in Table 2.

**Table 2:** Knowledge about net care, repair and exposure to information among household respondents, Ethiopia, 2015

Characteristics		Percentages (95% CI)
Knowledge about net care and repair (n = 1829)		
Handle the net with care		71.4% (69.2%, 73.5%)
Roll-up or tie-up when not in use		63.4% (61.3%, 65.4%)
Keep away from children		47.9% (45.5%, 50.2%)
Wash gently		36.2% (33.9%, 38.5%)
Keep away from flame/fire		35.0% (32.8%, 37.2%)
Keep away from pests and rodents		34.8% (32.7%, 40.0%)
Wash only when dirty		32.3% (30.2%, 34.6%)
Do not soil with food		19.7% (18.0%, 21.5%)
Inspect regularly for holes		17.7% (16.0%, 19.5%)
Repair small holes quickly		13.1% (11.6%, 14.7%)
At least five correct answers (n = 1761)		22.3% (20.4%, 24.3%)
Exposure to information on net care and repair (n = 1839)		
Received information in the last 6 months	Yes	24.6% (22.7%, 26.5%)
Content of information exposed to		
Hang-up your net (n = 452)	Yes	72.8% (68.6%, 77.1%)
Care for your net (n = 452)	Yes	56.6% (52.1%, 61.2%)
Repair your net (n = 452)	Yes	03.0% (01.5%, 05.1%)
Source of information (n = 450)		
HEWs/HDAs/other health workers		86.1% (82.5%, 89.0%)
Other sources (community leaders, radio, family or friend)		13.9% (11.0%, 17.5%)
Discussion on net care and repair with a family (n = 1839)	Yes	19.2% (17.5%, 21.1%)

### Perception about net care and repair

Almost all respondents reported believing that nets are valuable and 96.1% thought that they could help protect their family from malaria by taking care of their net. Although the majority (82.4%) responded that there are ways to make their net last longer, approximately half (47.2%) of respondents reported that a repaired net is not effective against mosquito bites. Almost 40% of respondents thought the repair of nets was not possible, and 32.5% did not have the confidence to make a repair. Almost a quarter (23.2%) of respondents indicated insufficient time to repair holes in their net and 47.0% did not think others in the community repaired holes in their nets either. Overall, 82.4% of the respondents had either a positive (i.e., perception score was above 0) or very positive (i.e., perception score was above 1) perception towards net care and repair (Table 3).

**Table 3:** Perceptions towards net care and repair among households, in Ethiopia, 2015

Variables (n = 1829)	Percentage (95% CI)
Mosquito nets are valuable	
Agree	99.9% (99.7%, 100%)
Neutral	0.02% (0.003%, 0.16%)
Disagree	0.05% (0.008%, 0.38%)
There are actions to make my net last long	
Agree	82.4% (80.5%, 84.2%)
Neutral	4.43% (3.5%, 5.6%)
Disagree	13.2% (11.7%, 14.8%)
It is not possible to repair holes in net	
Agree	39.6% (37.2%, 42.0%)
Neutral	3.68% (2.9%, 4.7%)
Disagree	56.8% (54.3%, 59.2%)
A repaired net can still be effective	
Agree	47.5% (45.1%, 49.8%)
Neutral	5.27% (2.9%, 4.7%)
Disagree	47.2% (44.9%, 49.6%)
Other people in this community fix holes in their net	
Agree	25.5% (23.5%, 27.6%)
Neutral	27.6% (25.6%, 29.7%)
Disagree	47.0% (44.7%, 49.2%)
Do not have time to repair holes	
Agree	23.2% (21.3%, 25.3%)
Neutral	2.46% (1.8%, 3.4%)
Disagree	74.3% (72.1%, 76.3%)
I can help protect my family from malaria by taking care of my net	
Agree	96.1% (95.0%, 97.0%)
Neutral	0.27% (0.09%, 0.08%)
Disagree	3.61% (2.8%, 4.7%)
I am confident I can repair holes immediately	
Agree	65.4% (63.2%, 67.6%)
Neutral	2.02% (1.4%, 2.9%)
Disagree	32.5% (30.5%, 34.7%)
Overall perception score	
Negative	17.6% (15.9%, 19.5%)
Positive	47.3% (44.9%, 49.7%)
Very positive	35.1% (32.9%, 37.4%)

Using ordinal logistic regression and overall perception levels as an outcome, the odds of positive perception around net care and repair did not increase in relation to exposure to information on net care and repair in the last 6 months, after controlling for socio-demographic variables. However, those who reported discussing net care and repair with their family were more likely to have a positive perception towards net care and repair (OR 1.47, 95% CI 1.14–1.89) compared to those who did not discuss net care and repair. Similarly, respondents with knowledge about net care and repair had 58% higher odds of having positive perception towards net care and repair (OR 1.58, 95% CI 1.23–2.02). The number of nets in the household did not show a statistically significant association with perception towards net care and repair (Table 4).

**Table 4:** Factors associated with household’s perception towards net care and repair, Ethiopia, 2015

	Perception towards net care and repair			Adjusted odds ratio (95% CI)	P value
	Negative	Positive	Very positive		
Knowledge on net care and repair					
Not adequate	273 (85.8%)	657 (80.6%)	434 (69.8%)		
Adequate knowledge	45 (14.2%)	159 (19.4%)	188 (30.3%)	*1.58 (1.23, 2.02)*	< 0.001
Exposure to information on net care and repair					
No	294 (91.5%)	751 (87.0%)	519 (80.9%)	1.00	
Yes	27 (8.5%)	113 (13.0%)	122 (19.1%)	1.24 (0.92, 1.65)	0.16
Discussion on net care and repair in the family					
No	289 (89.7%)	712 (82.4%)	476 (74.2%)	1.00	
Yes	33 (10.3%)	152 (17.6%)	166 (25.9%)	*1.47 (1.14, 1.89)*	< 0.001
Number of LLINs owned					
One	119 (36.9%)	270 (31.2%)	224 (35.0%)	1.00	
Two	126 (39.1%)	369 (42.7%)	257 (40.1%)	1.05 (0.83, 1.33)	0.70
Three or more	77 (24.0%)	225 (26.1%)	160 (24.9%)	1.10 (0.82, 1.49)	0.51
Gender					
Male	248 (77.26%)	687 (79.79%)	526 (81.93%)	1.00	
Female	73 (22.74%)	174 (20.21%)	116 (18.07%)	0.72 (0.56, 0.94)	0.06
Age (years)					
18–29	45 (13.98%)	108 (12.50%)	80 (12.46%)	1.00	
30–39	89 (27.64%)	264 (30.56%)	193 (30.06%)	0.93 (0.67, 1.30)	0.68
40–49	70 (21.74%)	217 (25.12%)	162 (25.23%)	0.91 (0.64, 1.29)	0.58
50–59	44 (13.66%)	113 (13.08%)	100 (15.58%)	1.06 (0.70, 1.58)	0.79
60+	74 (22.98%)	162 (18.75%)	107 (16.67%)	0.80 (0.55, 1.18)	0.27
Educational status of head of household					
No formal education	185 (57.63%)	457 (52.95%)	336 (52.34%)	1.00	
Primary (grade 1–6)	79 (24.61%)	223 (25.84%)	164 (25.55%)	0.98 (0.76, 1.26)	
Secondary (grade 7–8)	26 (8.10%)	75 (8.69%)	49 (7.63%)	1.05 (0.72, 1.52)	
More than secondary (≥ grade 9)	31 (9.66%)	108 (12.51%)	93 (14.49%)	1.08 (0.78, 1.49)	
Wealth index					
1st	73 (23.03%)	161 (18.74%)	128 (20.00%)	1.00	
2nd	61 (19.24%)	179 (20.84%)	123 (19.22%)	0.90 (0.67, 1.23)	0.52
3rd	74 (23.34%)	185 (21.54%)	106 (16.56%)	0.70 (0.51, 0.96)	0.03
4th	63 (19.87%)	182 (21.19%)	118 (18.44%)	0.81 (0.59, 1.13)	0.22
5th	46 (14.51%)	152 (17.69%)	165 (25.78%)	1.19 (0.85, 1.67)	0.30
Region					
Tigray	41 (12.73%)	212 (24.54%)	191 (29.75%)	1.00	
Amhara	89 (27.64%)	218 (25.23%)	175 (27.26%)	0.76 (0.58, 1.00)	0.05
Oromia	124 (38.51%)	257 (29.75%)	124 (19.31%)	0.49 (0.37, 0.65)	0.00
SNNP	68 (21.12%)	177 (20.49%)	152 (23.68%)	0.69 (0.52, 0.92)	0.01

The model was controlled for gender, age, educational status, wealth index, and region

Statistically significant association (P value < 0.05) are indicated in italics

## Discussion

In general, knowledge about net care and repair was low, as was those reporting to have received information or discussed net care and repair with their families, despite the recent behavioural change communication (BCC) campaign in the study area. This suggests that targeted BCC campaigns should be improved to focus on providing useful information on how to care for nets, thus increasing not only knowledge but also ability to care for and repair nets. However, most respondents did have positive perception towards net care and repair; respondents with knowledge about net care and repair and those who discussed net care and repair with their family were more likely to have positive perception. BCC campaigns should build on this observation and not only target knowledge and perception, but also provide specific guidance on how best to access resources and skills for the maintenance and repair of nets.

Authors of this study are not aware of any studies that captured an overall knowledge score on net care and repair; however, the three most common net care approaches mentioned in this study were also identified in studies elsewhere [19, 20, 25]. Careful handling of net and keeping nets out of children’s reach is a common technique used [25]. Repairing small holes quickly is likely the least cited technique in many places, as was found in this study [19]. While information regarding net care and repair would ideally be given out, this study found very few to have received any information. Results of this study suggest that increasing the amount and quality of information on net care and repair may increase positive perceptions, further reinforcing the idea that BCC could be an important tool; other studies have drawn similar conclusions [31].

A considerable proportion of respondents had positive perception towards net care and repair. Slightly higher level of positive perception towards net care was however observed across sub-Saharan Africa [19, 30]. This variation could be explained by socio-demographic characteristics such as gender or wealth [21], or perhaps because of study design. In addition, there was variation in the individual perception statements; while a majority of respondents believe they can take care of their nets and protect their family from malaria, not all respondents believed it was possible to repair a net or did not know how. Moreover, other studies have documented a perception that a torn net is no longer useful [23] and there is simply a preference for a new net once a net is torn [26]; both of these perceptions could be targeted by BCC/net distribution campaigns. In this context, social norms could also be targeted; many respondents thought that their neighbours did not repair nets and this may have influenced their perceptions. Other studies have found social norms to be an important motivator [21, 25].

## Limitations

In general, it is imperative to interpret the results of this study with some inherent caveats of the study design, such as the cross-sectional nature of the study, which may not allow establishing temporal relationship between perception and the exposure variables. In addition, asking questions about net repair 2 months after net distribution may not be ample time to assess repair practices, as many of the new nets are likely still intact. A third limitation relates to the division of labour within households; it is possible that those responsible for net care and repair might not be the head of households, or the adult resident who answered the questions. Thus, one reason why respondents in this study may have little to say about net care and repair is because the study failed to interview the person responsible for net care and repair. Fourth, as this study measured reported behaviour, and reported behaviour is sometimes subject to social desirability bias, especially given the data were collected shortly after the net distribution campaign where recipients could have been exposed to messages that influence their responses. A fifth limitation relates to whether knowledge and perception actually translate into practice; as this study only measured perceptions and knowledge, and not behaviour, it is possible that other factors are interacting to influence repair practices. Repair skill, self-efficacy, availability of repair resources, social norms, and people’s expectations about when another net distribution is likely to occur may also influence repair practices in a community. Lastly, it is unclear the extent to which the BCC campaign employed in 2015 actually addressed net care and repair in great detail; while most BCC campaigns focus on promoting net use, information on specific instructions for repairing or caring for nets is often not provided.

## Conclusions

The overall level of knowledge on net care and repair was low in the study areas and repairing holes in the nets was the least frequently mentioned method of net care approach. In addition, not enough information and technical assistance on net care and repair is reaching the communities. Although the overall positive perception level towards net care and repair was moderate, it was clear that barriers exist and should be addressed as part of national malaria control programme activities. The National Malaria Control Programme in Ethiopia should focus on providing resources and skills for repairing and caring for nets; in addition, it is imperative that programmes begin working to change social norms so that net owners feel that they are expected to care for and repair their nets. This will serve the dual purpose of increasing the longevity of nets, as well as building a cadre of community members skilled in net care and repair.

## Additional file

**Additional file 1.** Statement used to measure overall perception towards net care and repair.

## Abbreviations

ACIPH: Addis Continental Institute of Public Health
AOR: adjusted odds ratio
BCC: behaviour change communication
CI: confidence interval
EA: enumeration area
IRB: Institutional Review Board
LLIN: long-lasting insecticidal net
MIS: Malaria Indicator Survey
ODK: open data kit
PMI: US President’s Malaria Initiative
SNNP: Southern Nations Nationalities People
WHOPES: World Health Organization Pesticide Evaluation Scheme
