# Supplementary material for: Knowledge and perception towards net care and repair practice in Ethiopia
Source: Malar J. 2017 Oct 2;16:396. doi: 10.1186/s12936-017-2043-1 (PMC5625612; doi:10.1186/s12936-017-2043-1)
Supplement: Supplementary file 1 — Additional file 1. Statement used to measure overall perception towards net care and repair. [file 12936_2017_2043_MOESM1_ESM.doc]

**Additional file 1:**

**Statement used to measure overall perception towards net care and repair**

| - "Mosquito nets are valuable” |
| --- |
| - “There are actions I can take to make my net last longer" |
| - "It is not possible to repair holes in nets" |
| - "A repaired net can still be effective against mosquitoes" |
| - "Other people in this community fix holes in their mosquito nets" |
| - "I do not have time to repair a hole in my net" |
| - "I can help protect my family from malaria by taking care of my net" |
| - "I am confident I can repair holes immediately" |
